# Immobilization of *Acetobacter* sp. CCTCC M209061 for efficient asymmetric reduction of ketones and biocatalyst recycling

**DOI:** 10.1186/1475-2859-11-119

**Published:** 2012-09-04

**Authors:** Xiao-Hong Chen, Xiao-Ting Wang, Wen-Yong Lou, Ying Li, Hong Wu, Min-Hua Zong, Thomas J Smith, Xin-De Chen

**Affiliations:** 1 State Key Laboratory of Pulp and Paper Engineering, South China University of Technology, Guangzhou, 510640, People’s Republic of China; 2 Lab of Applied Biocatalysis, College of Light Industry and Food Science, South China University of Technology, Guangzhou, 510640, People’s Republic of China; 3 Biomedical Research Centre, Sheffield Hallam University, Owen Building, Howard Street, Sheffield, S1 1WB, UK; 4 Guangzhou Institute of Energy Conversion, Chinese Academy of Sciences, Guangzhou, 510640, People’s Republic of China

**Keywords:** Immobilization, Asymmetric reduction, *Acetobacter* sp. CCTCC M209061, Alginate-chitosan, Mass transfer

## Abstract

**Background:**

The bacterium *Acetobacter* sp. CCTCC M209061 is a promising whole-cell biocatalyst with exclusive anti-Prelog stereoselectivity for the reduction of prochiral ketones that can be used to make valuable chiral alcohols such as (*R*)-4-(trimethylsilyl)-3-butyn-2-ol. Although it has promising catalytic properties, its stability and reusability are relatively poor compared to other biocatalysts. Hence, we explored various materials for immobilizing the active cells, in order to improve the operational stability of biocatalyst.

**Results:**

It was found that Ca-alginate give the best immobilized biocatalyst, which was then coated with chitosan to further improve its mechanical strength and swelling-resistance properties. Conditions were optimized for formation of reusable immobilized beads which can be used for repeated batch asymmetric reduction of 4′-chloroacetophenone. The optimized immobilized biocatalyst was very promising, with a specific activity of 85% that of the free-cell biocatalyst (34.66 *μ*mol/min/g dw of cells for immobilized catalyst *vs* 40.54 *μ*mol/min/g for free cells in the asymmetric reduction of 4′-chloroacetophenone). The immobilized cells showed better thermal stability, pH stability, solvent tolerance and storability compared with free cells. After 25 cycles reaction, the immobilized beads still retained >50% catalytic activity, which was 3.5 times higher than degree of retention of activity by free cells reused in a similar way. The cells could be recultured in the beads to regain full activity and perform a further 25 cycles of the reduction reaction. The external mass transfer resistances were negligible as deduced from Damkohler modulus Da < <1, and internal mass transfer restriction affected the reduction action but was not the principal rate-controlling step according to effectiveness factors *η* < 1 and Thiele modulus 0.3<*∅* <1.

**Conclusions:**

Ca-alginate coated with chitosan is a highly effective material for immobilization of *Acetobacter* sp. CCTCC M209061 cells for repeated use in the asymmetric reduction of ketones. Only a small cost in terms of the slightly lower catalytic activity compared to free cells could give highly practicable immobilized biocatalyst.

## Background

Biocatalysis using enzymes, microorganisms and plant cells, has generated considerable interest for the synthesis of various enantiopure alcohols used as pharmaceutical and agrochemical intermediates due to its high efficiency, high enantioselectivity, mild reaction condition and low environmental pollution [1]. Whole-cell biocatalysts are particularly attractive for production of chiral alcohols by reduction of ketones because they contain all the necessary cofactors and the metabolic pathways for their regeneration. A variety of microorganisms have been used for the production of chiral alcohols such as yeasts [2], fungi [3,4] and bacteria [5]. However, little has been reported about the *Acetobacter* sp. for the asymmetric reduction of ketones. Recently, a novel bacterial strain, *Acetobacter* sp. CCTCC M209061, was isolated from Chinese kefir grains and characterized by our group as an active strain for the reduction of prochiral ketones [6]. This strain showed exclusive anti-Prelog stereoselectivity for the reduction of 4-(trimethylsilyl)-3-butyn-2-one to (*R*)-4-(trimethylsilyl)-3-butyn-2-ol, which is a key chiral intermediate for the synthesis of (*R*)-benzyl-4-hydroxyl-2-pentynoate, a potential therapeutic for Alzheimer’s disease. This strain can also be applied in the production of a range of enantiopure chiral alcohols that are valuable as building blocks for molecules required in many industries. Although *Acetobacter* sp. CCTCC M209061 can perform enantioselective biotransformations that have not been effectively performed by using other strains, several challenges remain before it can be used for industrial application. In particular, its stability and reusability were relatively poor compared to many other biocatalysts [7]. A range of immobilization technologies are commonly used for biocatalysts and can confer desirable features onto the catalyst [8]. Immobilization of whole cells and enzymes via entrapment in polymers such as agar [9], carrageenan [10], alginate gels [2,11], polyvinyl alcohol [12], and polyurethane foam [13] have been used very effectively for reduction of ketones with high enantioselectivity.

Acetophenone and its derivatives have been widely used as model substrates for screening of active microorganisms to catalyze reduction of ketones [14,15], and so the anti-Prelog asymmetric reduction of 4′-chloroacetophenone to (*R*)-1-(4-chlorophenyl)ethanol was chosen as a model reaction to investigate immobilization of the *Acetobacter* sp. CCTCC M209061 cells. Here we present the results of immobilization of cells in a range of matrices, the use of a complex entrapment-encapsulation method and optimization of the immobilization conditions with regard to stability and shelf life of the catalyst. In addition, the mass transfer limitations were also investigated based on established theory [16].

## Results and discussion

### Catalytic activity of free cells for the reduction of 4′-chloroacetophenone

**Figure 1:**
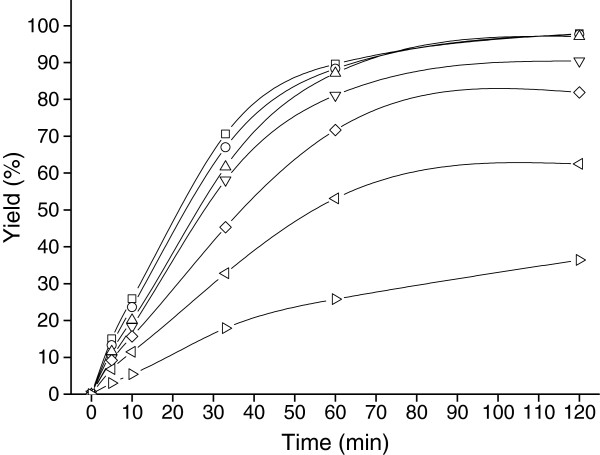
**Kinetic profile of the reduction of 4′-chloroacetophenone using free cells for biocatalyst re-use**. Reaction conditions: 8 mL TEA-HCl buffer (100 mmol/L, pH 5.0) containing 130 mmol/L of isopropanol and 5.0 mmol/L 4′-chloroacetophenone, 0.24 g wet cells, 30°C, 160 rpm. Cycle 1 (□), Cycle 2 (○), Cycle 3 (△), Cycle 4 (▽), Cycle 5 (◇), Cycle 6 (◁), Cycle 7(▷).

The catalytic activity of free cells was tested for recycled batch reduction of 4′-chloroacetophenone. As shown in Figure 1, the free cells could be reused three times with nearly 97% yield after 120 min reaction whereas only 36% yield was achieved at the seventh cycle; the formed product was confirmed to be (*R*)-1-(4-chlorophenyl)ethanol by ^1^ H NMR and optical rotation, and the product *e.e.* constantly remained more than 99%. The decrease in reduction activity was attributed to a combination inactivation of the biocatalyst and loss of biomass during its recovery between cycles. The difficulty in separating the cells from the reaction mixture and in recovering all the biocatalyst highlighted the need to develop an immobilization method to improve the practicability of this biocatalyst.

### Immobilization of cells with different materials

Before immobilization, cells of *Acetobacter* sp. CCTCC M209061 were grown into late log phase under the conditions previously shown to maximise enzyme activity [7]. The immobilized biocatalysts were directly used to catalyze the asymmetric reduction of 4′-chloroacetophenone [7]. In the case of cells adsorbed on activated-charcoal, diatomite or polyurethane foam, or entrapped in polyvinyl alcohol (PVA), gelatin etc., biocatalysts gave low yields in (*R*)-1-(4-chlorophenyl)ethanol or poor practicality (data not shown). Good yields were obtained with agar, *κ*-carrageenan and alginate matrices (Table 1). In order to allow comparison of catalyst effectiveness both in terms of productivity per mass of cells and productivity per mass of catalyst, two separate specific activities were calculated for each catalyst, with respect to dry weight (dw) of cells and wet weight (ww) of catalyst as follows [17]:

(1) $$\text{Specific}\;\text{activity}=\frac{\text{Yield}\left(\%\right)\times \left[\text{substrate}\right]\left(\mu mol\right)}{\text{Time}\left(\min\right)\times \left[\text{cells}\right]\left(g\;\text{dry}\;\text{wt}\right)or\left[\text{Cat}\right]\left(g\;\text{wet}\;\text{wt}\right)}$$

**Table 1:** **Catalytic performance of*Acetobacter*sp. CCTCC M209061 cells immobilized in various matrices**

**Immobilizing matrix**	**Time (min)**^**a**^	**Catalyst load (mg-dw/g)**	**v**_**0**_**(*μ*mol/min)**	**Specific activity (*μ*mol/min/g dw cells)**	**Specific activity (*μ*mol/min/g ww catalyst)**	**Yield (%)**	***e.e.*(%)**
None (free cells)	90	N/A^b^	1.62	40.54	N/A	97.0	>99%
Agar	120	5.79	1.08	27.09	0.16	95.1	>99%
*κ*-Carrageenan	120	5.56	1.18	29.61	0.16	97.3	>99%
Ca-alginate	90	7.81	1.42	34.78	0.27	97.5	>99%
Ba-alginate	90	7.35	1.41	34.79	0.26	97.3	>99%
Zn-alginate	120	7.69	1.04	25.96	0.20	98.5	>99%
Cu-alginate	120	7.46	0.67	16.68	0.12	73.4	>99%

Conditions: 8 mL TEA-HCl buffer (100 mmol/L, pH 5.0) containing 130 mmol/L of isopropanol and 5.0 mmol/L of 4′-chloroacetophenone, 0.24 g wet cells or immobilized matrix with 0.24 g wet cells, T = 30°C, 160 rpm.

^a^Reaction time to achieve the maximum yield.

^b^N/A, not applicable.

The Cu-alginate immobilization system gave lowest specific activities, probably because the Cu^2+^ ions were toxic to the cells or inhibited biocatalytic activity. Zn-alginate gave soft and weak beads which swelled and became damaged after only four cycles of use. In the case of agar and *κ*-carrageenan, where an elevated temperature (45–50°C) is used to keep the matrices in the sol state when the cells are added, the relatively low biocatalyst activities may be due to heat inactivation of the cells during immobilization. The best results were obtained with the Ca-alginate and Ba-alginate, which are prepared at ambient temperature and gave strong gels that were biocompatible and allowed good catalytic activity (Table 1).

### The optimization for complex immobilization

Attempts to use the Ca-alginate immobilized *Acetobacter* sp. CCTCC M209061 as a recyclable biocatalyst revealed that mechanical strength and swelling behavior of Ca-alginate beads were not sufficient. In order to overcome these limitations, a coating of chitosan was used to interact electrostatically with the alginate to produce a compact chitosan membrane at the surface of alginate beads [18].

The conditions for immobilizing the cells using Ca-alginate and chitosan were optimized singly as detailed in Table 2. The concentration of alginate was varied between 1.5 and 4.0% (w/v), because below 1.5% the gel strength was very weak and the shape of beads was not spherical. There was no substantial difference in the reduction activity of cells immobilized at different alginate concentrations, which can be explained by the negligible effect of alginate concentration on diffusion of small compounds into the particles [19]. However, the effect of the alginate concentration on the mechanical properties of the immobilized catalyst was substantial. Sodium alginate concentration above 4.0% led to a very viscous solution, difficulties in mixing completely without the formation of bubbles, and difficulties in producing beads with a spherical shape [20]. 2.0%-3.0% sodium alginate concentration resulted in spherical beads and strong gel and so 2.5% was chosen as the best concentration to immobilize the cells.

**Table 2:** Optimization of the Ca-alginate/chitosan hybrid immobilization

**Immobilization conditions**^**a**^	**Values**	**v**_**0**_**(*μ*mol/min)**^**b**^	**Specific activity (*μ*mol/min/g dw cells)**^**c**^	**Specific activity (*μ*mol/min/g ww catalyst)**^**c**^	**Reuse cycles**^**d**^	***e.e.*(%)**
Alginate concentration	1.5	0.96	24.10	0.45	6	>99%
(% w/v)	2.0	0.97	24.18	0.26	8	>99%
	3.0	0.95	23.80	0.18	8	>99%
	4.0	0.95	23.68	0.17	8	>99%
Chitosan pH	3.0	0.00	0.00	0.00	0	>99%
	3.5	0.00	0.00	0.00	0	>99%
	4.0	0.98	24.53	0.25	8	>99%
	4.5	1.32	33.10	0.32	14	>99%
	5.0	1.37	34.30	0.33	14	>99%
Chitosan concentration	0	1.39	34.78	0.31	10	>99%
(% w/v)	0.3	1.39	34.65	0.32	12	>99%
	0.6	1.38	34.38	0.32	14	>99%
	0.9	1.38	34.47	0.33	14	>99%
	1.2	1.36	34.03	0.33	14	>99%
	1.5	1.36	34.10	0.33	14	>99%
Coating time	5	1.38	34.53	0.32	10	>99%
(min)	10	1.38	34.38	0.33	14	>99%
	15	1.37	34.20	0.34	14	>99%
	20	1.36	33.73	0.35	14	>99%
	30	1.34	34.30	0.37	14	>99%
Cell loading	6.90	1.10	33.24	0.23	12	>99%
(mg-dw cells/	10.34	1.62	33.46	0.35	16	>99%
g-ww catalyst)	13.79	2.18	33.56	0.46	21	>99%
	17.24	2.79	34.66	0.60	25	>99%
	20.69	3.05	32.04	0.66	25	>99%

Conditions: 8 mL TEA-HCl buffer (100 mmol/L, pH 5.0) containing 130 mmol/L of isopropanol and 5.0 mmol/L of 4′-chloroacetophenone, 2.76 g immobilized beads, T = 30°C, 160 rpm.

^a^ Immobilization perameters were initially: chitosan pH, 3.89; chitosan concentration 1.0% w/v; coating time 15 min; cell loading 8.82 (mg-dw cells/g-ww cat). Individual immobilization perameters were then optimized in the order shown in the table. As each parameter was optimized, it was fixed at the optimal value indicate during optimization of subsequent parameters. Values chosen as optimal are shown in bold in the table. During optimization of alginate concentration it appeared that the best performance was between 2 and 3% wt/v alginate and so a concentration of 2.5% was chosen as optimal.

^b^ The reaction rate during the first cycle.

^c^ The specific activity during the first cycle.

^d^ The number of cycles achieved whilst maintaining the yield at 120 min reaction time at ≥50%.

The chitosan coating on the beads, which was produced via a method based on that of Lu et al. [21], improved the surface properties and mechanical strength of the beads, although it is clear from data in Table 2 that the catalytic activity achieved was substantially less than that of free whole-cell catalyst or when the chitosan coating was omitted (Table 1). It was reasoned that the low pH of the chitosan solution (pH 3.9) might contribute to this low activity by inactivating the reduction activity of the cells. It was found that the activity was greatly increased with increasing pH of the chitosan solution from 3.5 to 5.0. The immobilized cells were completely deactivated at pH ≤3.5, but no deactivation was observed at pH 4.5-5.0. This might be related to the physiological properties of *Acetobacter* sp. CCTCC M209061, which shows optimal growth and reduction activity at slightly acidic pH (5.0-6.0). The physical properties of the polymers may also play a role. Chitosan is a polycationic polymer at pH 3.5-5.0 due to its pKa (6.2–7.0) and alginate is polyanionic polymer (pKa 3.38-3.65) [22]. The increase of pH from 3.5 to 5.0 would be expected to decrease the interaction between the two polymers by decreasing the degree of amino group protonation and thus increase the diffusion of chitosan into the alginate beads [23]. Here, the chitosan membrane formed from chitosan solution at pH 5.0 yielded beads with the best mechanical strength and high reduction activity (v_0_ = 1.37 *μ*mol/min; specific activity = 34.36 *μ*mol/min/g; which kept ≥ 50% activity over 14 cycles).

The concentration of chitosan in the solution used for coating the beads did not greatly affect the activity of the immobilized biocatalyst, but the mechanical strength was influenced markedly. An increase in chitosan concentration up to 0.9% led to higher mechanical strength of the beads and better resistance to swelling during use. When the chitosan concentration was above 1.2%, a thinner and denser membrane was formed with lower permeability to further infiltration of chitosan into the beads and lower mechanical strength. The reason might be that the compact membrane structure that formed quickly at such high chitosan concentration hindered permeation of chitosan into particles to form a thicker and stronger membrane [24]. The small decline in reduction activity as the chitosan concentration increased may be related to the thickness and structure of membranes and is in agreement with data from previous studies [25]. A chitosan concentration of 0.9% was concluded to be the best to give a combination of high reduction activity, mechanical strength and recyclability. The effectiveness of the chitosan coating can be appreciated by noting that approximately 50% of the beads were found to swell and rupture without chitosan coating after only eight reaction cycles, whereas no rupture and little swelling was found after 14 cycles of reaction when beads coated with chitosan under these conditions.

The coating time was varied between 5 and 30 min to see whether a longer coating time would afford a stronger, thicker coating and inhibit swelling of the beads during use by allowing more chitosan to permeate into the alginate component of the beads [26]. A longer exposure to the chitosan solution might also improve the strength and swelling resistance of the beads by favoring formation of more cross-linking interactions within the polymer network [18]. Within the range of coating time shown in Table 2, there was an increase in the recyclability of the beads up to 10 min coating time and the reduction activity of immobilized biocatalyst was little affected by the coating time within the range tested. The last observation may be due to the negligible diffusion resistance towards small molecular substrates into the particles as reported by Qi et al. [27]. The diameter of the beads decreased with increasing coating time, which was attributed to enwrapping of the alginate beads by the chitosan thus preventing their swelling and uptake of water [28]. As previously observed by Wang et al. [28], there appeared to be little increase in membrane thickness beyond 20 min coating time, therefore prolonging coating time would not give further benefit and 20 min was chosen as the optimal time for coating with chitosan.

As the cell loading of the beads was increased from 6.90 to 17.24 mg-dw cell/g-ww beads, the activity per g of catalyst increased (because the catalysts contained more cells per g) whilst the specific activity per g of cells increased relatively little. When the loading was increased further, to 20.69 mg-dw cells/g of beads, there was a reduction in the effectiveness of the immobilized catalyst, perhaps because penetration of substrates was inhibited when the beads were overloaded with cells [29]. Excessively high cell loading also caused increased cell leakage and decreased mechanical strength of the beads. Therefore, 17.24 mg-dw cell/g-ww catalyst was chosen as the best value for the immobilization.

The optimized conditions for immobilization of *Acetobacter* sp. CCTCC M209061 in alginate-chitosan beads were used for further characterization of the immobilized biocatalyst described below.

### The stability of biocatalysts

#### Thermal and pH stability

**Figure 2:**
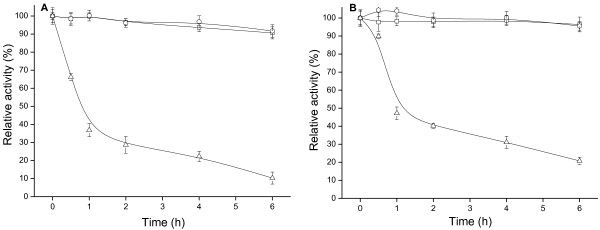
**Thermal stability of free cells (A) and alginate-chitosan immobilized cells (B).** 30°C (□), 40°C (○), 50°C (△). Residual activities were measured at pH 5.0 as indicated in the Experimental Section. Error bars show standard deviation from separate experiments conducted in duplicate.

**Figure 3:**
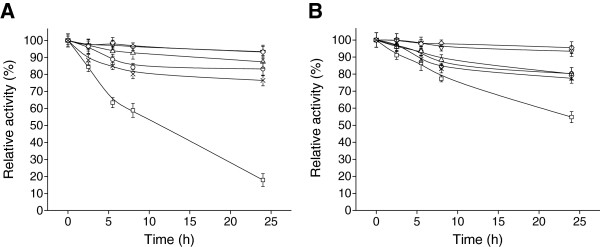
**pH stability of free cells (A) and alginate chitosan immobilized cells (B).** pH 3(□), pH 4 (○), pH 5 (△), pH 6(▽), pH 7 (◇), pH 8 (×). Residual activities were measured at 30°C. Error bars show standard deviation from separate experiments conducted in duplicate.

The optimized alginate-chitosan immobilized biocatalyst prepared was characterized with respect to its thermal (Figure 2) and pH (Figure 3) stability, in comparison with free cells of *Acetobacter* sp. CCTCC M209061. The immobilized biocatalyst and free cells showed retention of >90% of their activity during 24 h of incubation at 30 or 40°C. However, when the temperature was raised to 50°C, there was significant decrease in the activity of the immobilized and free cells over a 6 h period. And the immobilized biocatalyst (Figure 2B) provided a slight advantage in thermal stability over the free cells (Figure 2A). At 60°C, both were totally deactivated after 1 h (data not shown).

Immobilized biocatalyst and free cells retained >80% of their initial activity after incubation for up to 24 h between pH 4.0 and pH 8.0 (Figure 3). However, at pH 3.0, considerable inactivation was observed over a 24 h period, with free (Figure 3A) and immobilized (Figure 3B) cells losing 82% and 45%, respectively, of their initial activity. It is clear that immobilization with alginate–chitosan beads is effective in increasing the pH stability of the biocatalyst, probably owing to the micro-environment provided by the gel networks which may shield cells from the effects of H^+^ ions.

#### Tolerance to solvents

**Figure 4:**
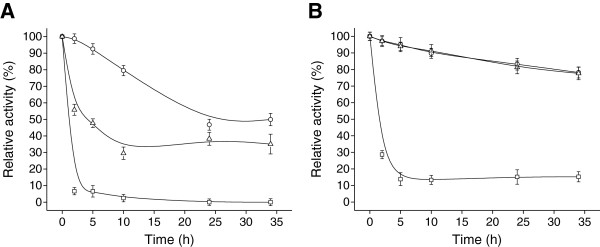
**Solvent tolerances of free cells (A) and alginate-chitosan immobilized cells (B).** Ethyl acetate (□), *n*-hexane (○), C_4_MIM·PF_6_ (△). Residual activities of the biocatalysts were measured in aqueous buffer after separating from the solvent, as indicated in the Experimental Section. Error bars show standard deviation from separate experiments conducted in duplicate.

Two organic solvents with different Lg*P* and the hydrophobic ionic liquid 1-butyl-3-methylimidazolium hexafluorophosphate (C_4_MIM·PF_6_) were chosen in order to investigate the solvent tolerance of free and immobilized cells. The immobilized cells showed markedly better tolerance to all three solvent systems was markedly better than the free cells (Figure 4). Both biocatalysts were quickly deactivated by ethyl acetate (Lg*P* 0.68), which may be due to the accumulation of solvents in the membrane lead to the destruction of membrane integrity and hence disturb the functions of membrane [30]. It is well described that solvents with Lg*P* < 4.5 are toxics for microbial cells [31]. Nonetheless, whilst 5 h of incubation in ethyl acetate almost completely deactivated the free cells, the immobilized biocatalyst retained 15% of its initial activity even after 34 h in this solvent. After 34 h exposing to hexane, which has a higher Lg*P* (3.5), the activity of the free cells decreased to 50% of the initial value, whilst the immobilized biocatalyst still retained 80% of its initial activity. Similarly, the immobilized cells retained about 80% of its initial activity after 34 h incubation in C_4_MIM·PF_6_, which was much higher than the free cells. Obviously, the immobilized biocatalyst showed resistance to inactivation compared to the free one in all three solvent systems. Additionally, the relatively better stability of *Acetobacter* sp. CCTCC M209061 cells in the presence of C_4_MIM·PF_6_ might be due to the better biocompatibility of the ionic liquid with the microbial cells, which was in good agreement with the previous reports that C_4_MIM·PF_6_ had the relatively lower toxicity to *Rhodotorula* sp. AS2.2241 cells [2] and recombinant *Escherichia coli*[32]. Furthermore, the hydrophobic ionic liquid-containing biphasic system can effectively eliminate the inhibitory and toxic effects of substrate and/or product on microbial cells and greatly improve the biotransformation with microbial cells, and thus show promising potential for biocatalytic reduction of prochiral ketones to chiral alcohols [32]. Therefore, the use of various ionic liquids including C_4_MIM·PF_6_ for the biotransformations with whole cells of *Acetobacter* sp. CCTCC M209061 is now underway in our laboratory.

#### Storability of biocatalysts

**Figure 5:**
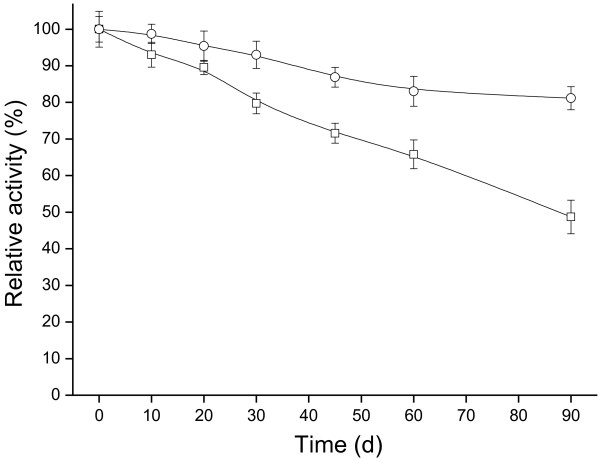
**Storage stability of free-cells and immobilized biocatalysts at 4°C.** Samples were withdrawn at various time intervals for determination reduction activities at 30°C using the 4′-chloroacetophenone substrate. Free cells (□), alginate-chitosan immobilized cells (○). Error bars show standard deviation from separate experiments conducted in duplicate.

The immobilized cells showed superior retention of activity compared to free cells during storage at 4°C for up to 90 days (Figure 5). During this period, the alginate-chitosan immobilized cells kept approximately 80% of their initial activity respectively while free cells retained only 50% activity. Hence, immobilization *Acetobacter* sp. CCTCC M209061 cells resulted in a biocatalyst with excellent storage properties.

### Recyclability of immobilized cells

**Figure 6:**
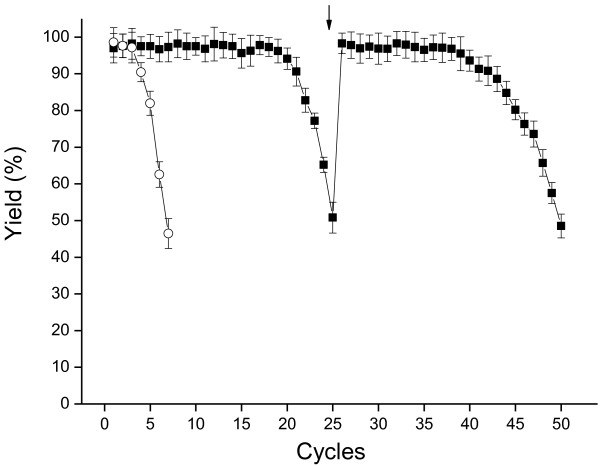
**The repeated asymmetric reduction of 4′-chloroacetophenone with the complex-immobilized cells and free cells.** Repeated actions were carried out with the free cells (○) and the complex immobilized cells (▪) in 8 mL TEA-HCl buffer (100 mmol/L, pH 5.0) containing 130 mmol/L of isopropanol and 5.0 mmol/L of 4′-chloroacetophenone, 4.7 g ww immobilized biocatalyst or 0.48 g wet free cells, 30°C, 160 rpm, reaction time for each batch was 60 min. Arrow: recultivation of 24 h was carried out as described in Materials and methods.

As shown in Figure 6, the reusability of alginate-chitosan immobilized *Acetobacter* sp. CCTCC M209061 cells was much better than the free cells during successive cycles of asymmetric reduction of 4′-chloroacetophenone. The yield of alcohol dropped to about 46% after seven 60-min cycles of reaction with the free-cell biocatalyst; however, when the immobilized biocatalyst was used, the yield maintained at 51% even after 25 cycles of reaction and the beads did not rupture. Consequently, it is clear that the immobilization of the *Acetobacter* sp. CCTCC M209061 cells offers significant advantages in terms of increasing the process stability of the cells, which is likely to make their commercial implementation simpler and more cost-effective. Previously, the reactivation of deactivated immobilized yeast cells has reported to allow their long term reuse for stereoselective reduction of ethyl 3-oxo esters [33,34]. Here, it was found that the deactivated immobilized cells could be reactivated by means of a 24 h recultivation step (shown by the arrow on Figure 6), which resulted recovery of reduction activity to a level that was slightly higher than the fresh immobilized cells and similar subsequent yields during successive cycles of reaction. Consistent with these observations, the cell loading within the beads increased about 10% after the 24 h recultivation step.

### Mass transfer consideration

#### Determination of external mass transfer parameters

**Figure 7:**
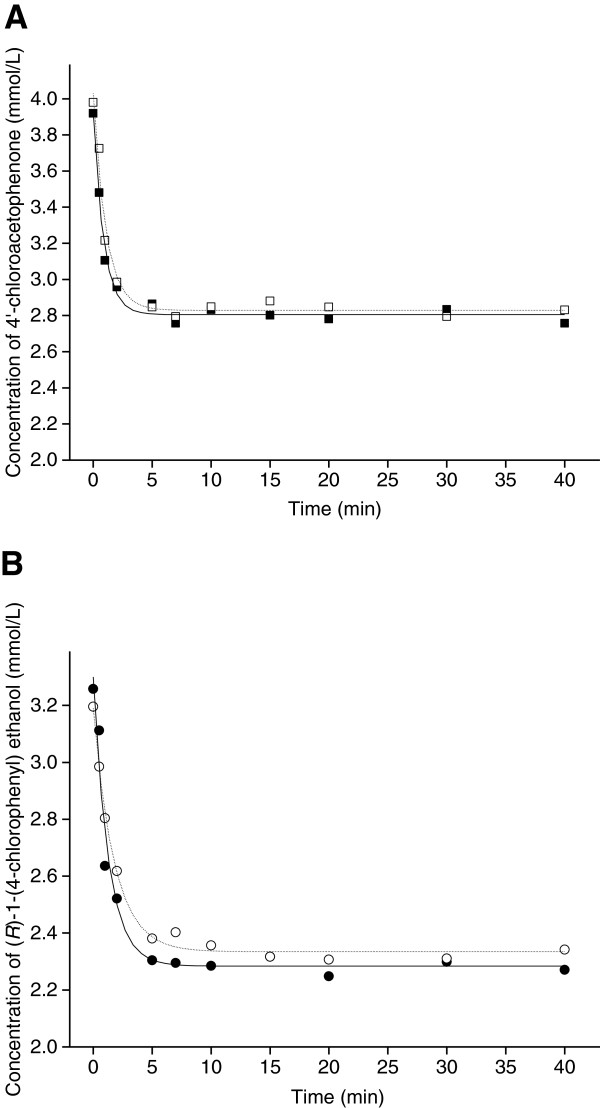
**Mass transfer of 4′-chloroacetophenone (A) and (*R*)-1-(4-chlorophenyl)ethanol (B).** From bulk solution into Ca-alginate (open symbols) and alginate-chitosan beads (solid symbols). Symbols represent observed concentrations obtained from experiments. Curves represent predicted concentrations from the best fit of equation (2) for these experimental data.

The external mass transfer, from the bulk solution into the beads, was studied experimentally for 4′-chloroacetophenone and (*R*)-1-(4-chlorophenyl)ethanol by using beads of calcium alginate (0.23 cm diameter) and alginate-chitosan (0.20 cm diameter) to which no cells were added. The variation of reagent concentrations in the bulk solution was fitted to Equation (2): [16]

(2) $$C\left(t\right)=Ae^{\left(-kt\right)}+C_{e}$$

where C (t) is the reagent concentration in the liquid at different diffusion times (mmol/L), k is mass transfer apparent constant (min^-1^), C_e_ is the reagent concentration (mmol/L) whent→∞. The experimental data for diffusion of 4′-chloroacetophenone and (*R*)-1-(4-chlorophenyl)ethanol into the beads are shown in Figure 7. And the external mass transfer parameters shown in Table 3 were calculated as follows. The mass transfer apparent constants (k) were calculated by fitting equation (2) to the experimental data (best-fit lines are shown in Figure 7) and then the liquid–solid mass transfer efficient K_L_(cm/min) can be determined by $K_{L}=k/a_{s}$, where *α*_*s*_ is the specific surface area for mass transfer (cm^-1^), which for the spherical beads is defined as 3/R(where R is the radius of the beads) [16].

**Table 3:** External and internal mass transfer parameters

	**External parameters**				**Internal parameters**			
	**ν**_**0**_**(*μ*mol/min)**	**K**_**L**_**× 10**^**2**^**(cm/min)**	**N**_**1**_**(mmol/min)**	**Da × 10**^**4**^	**k**_**1**_**× 10**^**2**^ **(min**^**-1**^**)**	***η***_***i***_	***ϕ***	**D**_**eff**_**× 10**^**4**^**(cm**^**2**^**/min)**
Free cells	1.62				5.23			
Ca-alginate	1.39	4.34	5.66	2.46	5.44	0.86	0.54	2.70
Alginate-chitosan	1.34	3.56	4.64	2.89	5.27	0.83	0.62	1.54

The mass transfer rate N_1_ from the bulk solution into the support beads is given by Equation (3):

(3) $$N_{1}=K_{L}\cdot a_{S}\cdot \left(C_{1}-{C_{1}}_{S}\right)$$

where N_1_ is the volumetric mass transfer rate (mM/min), (C_1_-C_1s_) is the concentration difference between the bulk solution and support external surface (mmol/L).

Then the observable Damkohler modulus (Da) for external mass transfer was then obtained from Equation (4) [35]:

(4) $$Da=\frac{v_{\text{obs}}}{K_{L}\cdot a_{s}\cdot C_{1}}=\frac{v_{\text{obs}}}{k\cdot C_{1}}$$

where **ν**_*obs*_ is the observed reaction rate for immobilized biocatalysts. From these data it can be seen that the *K*_*L*_ and *N*_1_ of Ca-alginate beads were close to chitosan coated beads which indicate comparable effective external mass transfer in both bead systems. In addition, the K_L_ values of the substrate 4′-chloroacetophenone in Ca-alginate and alginate-chitosan beads (4.34 and 3.56 × 10^-2^ cm/min, respectively) were greater than the corresponding values for the product (*R*)-1-(4-chlorophenyl)ethanol (2.57 and 1.87 × 10^-2^ cm/min, respectively), indicating that mass transfer efficiency was greater for substrate than for product. This result might be explained by changes in molecular structure and surface charges upon reduction of ketone group. Values of Da for the Ca-alginate and alginate-chitosan matrices were both < <1, which implies that C_1s_ ≈ C_1_ and so the external mass transfer resistances can be neglected.

#### Determination of internal mass transfer parameters

According to above results, the effect of external mass transfer was not significant, and in order to measure the effect of internal mass transfer resistance on reduction activity of immobilized cells, the effectiveness factor *η*_*i*_, which is defined as the ratio between the observed reaction rate for the immobilized biocatalysts ($v_{0,i}$) and the reaction rate for free cells ($v_{0,f}$) was used [16]:

(5) $$\eta_{i}=\frac{v_{0,i}}{v_{0,f}}$$

Evidently, *η*_*i*_=1 would indicate a negligible internal mass transfer resistance and *η*_*i*_<1 would show that the internal mass transfer is significant in limiting the reaction rate.

A dimensional parameter known as Thiele modulus (*ϕ*) can also be used to indicate the effect of mass transfer resistance on the overall reaction rate [17]. For a first order reaction and spherical biocatalyst, the Thiele modulus (*ϕ*) can be calculated according to Equation (6):

(6) $$\phi =\frac{R}{3}\sqrt{\frac{k_{1}}{D_{\text{eff}}}}$$

where R is the radius of biocatalyst bead (cm); D_eff_ is effective diffusion coefficient (cm^2^/min); k_1_ is the kinetic constant (min^-1^) .

The reaction yield was measured as a function of time for the two immobilized biocatalyst systems and the free cells over a period of 120 min. These experimental data showed that the yield at time t, Y(t) could be fitted to the first order model as Equation (7) [17]:

(7) $$Y\left(t\right)=B\left(1-e^{\left(-k_{1}t\right)}\right)$$

Thus, the kinetic constant (k_1_) was calculated from the experimental data using ORIGIN 7.5 (OriginLab, 2005). By assuming that the actual reduction rate (v) equals the diffusion rate, the effectiveness factor *η*_*i*_ can be expressed in terms of the Thiele modulus *ϕ*[16,36,37].

(8) $$\eta_{i}=\frac{1}{\phi }\left(\frac{1}{\tanh3\phi }-\frac{1}{3\phi }\right)$$

The values of parameters for internal mass transfer detemined from the experimental data are shown in Table 3. For the immobilized biocatalysts, *η*_*i*_<1 and *ϕ* >0.3, indicating that the reduction of 4′-chloroacetophenone is affected by internal mass transfer resistance [19,38]. The Thiele modulus *ϕ* increases with increasing effect of intraparticle diffusion on the reaction and it is considered that the effect of intraparticle diffusion on reaction rate is large when *ϕ* > 5 [39]. Here, the values of *ϕ* were < 1 and so the rate of the reduction was limited by internal mass transfer only to a moderate extent and the reaction showed effect of intraparticle diffusion and chemical reaction on the overall reduction process. The values of D_eff_ in Ca-alginate and alginate-chitosan beads (2.70 × 10^-4^ cm^2^/min and 1.54 × 10^-4^ cm^2^/min, respectively) were similar to the D_eff_ value of acetophenone in water added with alcohols [40]. In addition, compared with the Ca-alginate beads, alginate-chitosan biocatalyst showed slightly lower values of *η*_*i*_ and D_eff_ indicating that coated with chitosan increased resistance for diffusion.

**Figure 8:**
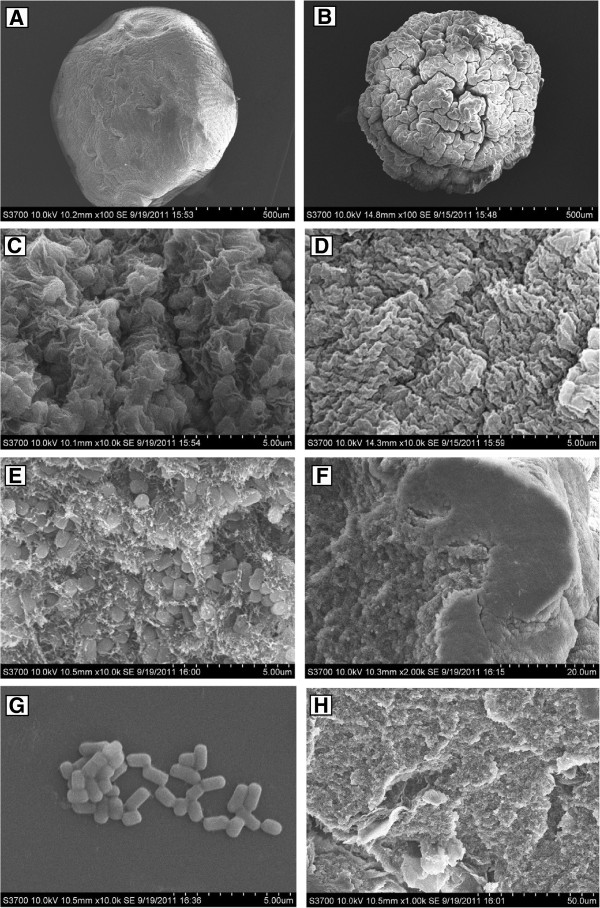
**Scanning electron micrographs of the Ca-alginate and alginate–chitosan beads containing *Acetobacter *cells.** The surface view of Ca-alginate (A, ×100; C, ×10.0 k) and chitosan-coated beads (B, ×100; D, ×10.0 k); the inside view of Ca-alginate (E, ×10.0 k); cross-section of at the edge of an alginate-chitosan bead showing the peripheral membrane structure (F, ×2.00 k); free cells (G, ×10.0 k) and inside view of alginate-chitosan (H, ×1.00 k); Voltage: 10.0 kV.

Further insight into these results was obtained by performing scanning electron microgroscopy (SEM) of the surface and inner structures of Ca-alginate and chitosan-coated alginate beads (Figure 8). The freeze drying procedure used during preparation of the beads for SEM resulted in shrinkage of both types of bead to about half their original size and the appearance of corrugations on the surface of the chitosan-coated beads (Figure 8A, B), which was similar to the previous observations of Azarnia et al. [22]. Nonetheless, the overall and micro (detailed) structures can be seen very clearly in the micrographs. At high magnification, the surface of the chitosan-coated beads (Figure 8D) was more rough and compact surface compared to the Ca-alginate beads (Figure 8C). The strong electrostatic interaction between chitosan and alginate may contribute to this difference. In both cases, when the beads were cut open the resulting inside views of the beads allowed the immobilized *Acetobacter* cells to be seen (Figure 8E,H). The structure of the peripheral membrane of the chitosan-coated bead was more compact than that of center part, presumably owing to the electrostatic interactions between carboxyl groups of alginate and amino groups of chitosan (Figure 8F). A similar membrane structure was observed previously due to cross-linking between sodium tripolyphosphate and chitosan [41]. The compact alginate-chitosan membrane may explain the stronger mechanical properties, lower tendency to swell, and lower values of the effectiveness factor *η*_*i*_ and D_eff_ of alginate-chitosan beads compared to Ca-alginate beads.

## Conclusions

It was shown that Ca-alginate is a highly effective material for entrapment of *Acetobacter* sp. CCTCC M209061 cells for repeated use in the asymmetric reduction of ketones. It was further shown that the alginate beads containing the cells can be made mechanically stronger and more resistant to swelling by coating with chitosan. Entrapment and coating have only a small cost in terms of the slightly lower catalytic activity compared to free cells; consequently the immobilized biocatalyst is highly practicable. Using the alginate-chitosan immobilization system, the stereoselective reduction reaction could be carried out repeatedly (up to 25 cycles) and a 24 h period of recultivation fully restored activity and permitted reuse in a further 25 cycles of the reaction. The reduction of 4′-chloroacetophenone to (*R*)-1-(4-chlorophenyl)ethanol catalyzed by the different immobilized biocatalysts gave high enantioselectivity (*e.e.* > 99%) under all the conditions tested. The mass transfer experiment indicated that the effect of external mass transfer limitation could be neglected and the Thiele modulus, 0.3<*ϕ*<1 indicated that the internal mass transfer restriction affected the reduction action but was not the principal rate-controlling step.

## Methods

### Biological and chemical materials

*Acetobacter* sp. CCTCC M209061 was isolated previously from Chinese kefir grains and maintained in our laboratory [6].

4′-Chloroacetophenone (97% purity), (*R*)-1-(4-chlorophenyl)ethanol (>99% purity), (*S*)-1-(4-chlorophenyl)ethanol (>99% purity), were purchased from Sigma–Aldrich (USA). Sodium alginate was purchased from Tianjin FUCHEN. Chitosan (with >95% deacetylation) was obtained from Sanland (USA). All other reagents and solvents were of analytical grade and were used without further purification.

### Culture conditions

*Acetobacter* sp. CCTCC M209061 was cultured in the medium containing 8.3 g/L glucose, 2.5 g/L fructose, 80 g/L soy peptone and 0.09 g/L MnSO_4_·H_2_O, pH was adjusted to 5.7. The medium was sterilized by autoclaving at 121°C for 20 min [7].

Cultivation of *Acetobacter* sp. CCTCC M209061 was performed in 250 mL Erlenmeyer flasks containing 50 mL of the above medium, which was inoculated with 10% (v/v) fresh cells. Cultivation was performed in a rotary shaker (WiseCube, Korea) at 30°C and 80 rpm. Wet cells were harvested between 30–33 h by centrifugation (4°C, 8000 rpm for 10 min), washed twice with sterilized water before using for the reduction activity assay and immobilization.

### Immobilization of *Acetobacter* sp. CCTCC M209061

Entrapment in agar or *κ*-carrageenan: 1.2 g wet cells were completely mixed with 20 mL of previously sterilized 4% (w/v) agar or 3% (w/v) *κ-*carrageenan at 45–50°C. Agar containing cells was allowed to set at room temperature; when the cells were mixed with *κ-*carrageenan, the mixture was placed at 4°C for 30 min before immersing in 0.3 mol/L KCl at 4°C for 4 h. The gel was cut in small cubes (0.3 cm × 0.3 cm × 0.3 cm), washed with TEA-HCl buffer (100 mmol/L, pH 5.0, 130 mmol/L isopropanol) and stored at 4°C until use.

Entrapment in alginate: 1.2 g wet cells were completely mixed with 20 mL previously sterilized 2% (w/v) alginate sodium. Then, the mixture was added drop-wise from a syringe to a gently stirred solution of 0.2 mol/L CaCl_2_, ZnCl_2_, CuCl_2_ or BaCl_2_. The resulting gel beads (diameters between 2.5 and 3.0 mm) were hardened at room temperature for 2 h and then 4°C for 10 h. After this, the wet beads were filtrated, washed and stored in TEA-HCl buffer (100 mmol/L, pH 5.0, 130 mmol/L isopropanol) at 4°C until use.

Coating with chitosan: The alginate-chitosan beads were prepared by modifying the methods reported previously [21,42]. The mixture of alginate (at concentration ranging from 1.5% to 4.0% (w/v)) and cells (4–12 g-dw/L) was added drop-wise to a gently stirred solution of 0.2 mol/L CaCl_2_, and the beads were hardened for 30 min at room temperature. The gel beads thus formed were then transferred to chitosan solution (0.3%-1.5% (w/v); pH 3.5-5.0) for 5–30 min to form the membrane structure. The coated beads (0.2-0.3 cm diameter) were collected by filtration and washed with sterilized water to remove residual chitosan. The resulting beads were stored in TEA-HCl buffer (100 mmol/L, pH 5.0, 130 mmol/L isopropanol, 5 mmol/L CaCl_2_) at 4°C until use.

In order to determine the cell loading of the beads, 20 gel beads were collected in a test tube. Sodium citrate solution (55 mmol/L, 6 mL) was added to liquefied alginate matrix and then the membrane was perforated to release cells. The solution was diluted as required and the absorbance was measured at 420 nm and converted to cell concentration (g-dw cell/L) by using a calibration curve.

### Reduction of 4′-chloroacetophenone (reduction activity assay) and biocatalyst recycling

The reduction was performed in a 50-mL Erlenmeyer flask with 8 mL TEA-HCl buffer (100 mmol/L, pH 5.0) containing 130 mmol/L of isopropanol (the source of reducing equivalents) and 5.0 mmol/L of 4′-chloroacetophenone, capped with a septum and pre-incubated in a shaking incubator at 30°C and 160 rpm for 10 min. Then, the free or immobilized cells were added into flask to initiate the reaction. Samples (50 *μ*L) were withdrawn at specified time intervals. The product and the residual substrate were extracted twice with isopropyl ether (100 *μ*L) containing 5.0 mmol/L 3′-methoxyacetophenone (internal standard) prior to GC analysis.

After one cycle of reduction using immobilized cells, the liquid was collected by filtration and extracted twice with isopropyl ether; solvent was removed under reduced pressure. The product was purified by flash column chromatography though a small pad of silica gel using n-hexane/ethyl acetate (9:1) as solvent. And the biocatalyst was washed with TEA–HCl buffer (pH 5.0) three times. Then fresh substrate solution was added to start a new batch of the reaction. Free cells were recycled by centrifugation after each batch. Other conditions were the same as those used with immobilized cells.

When reduction activity of the immobilized catalyst became insufficient after successive repeated batches, the reaction mixture was removed by filtration, and the biocatalyst was washed with sterilized water three times. Then fresh medium which was the same as used for the cultivation except for the addition of 0.3% (w/v) CaCl_2_ was added to recultivate those biocatalysts at 30°C, 80 rpm for 24 h.

### Stability of biocatalysts

Temperature Stability: 0.6 g wet free cells or 6.8 g immobilized beads (approximate 0.10 g-dw cells) in TEA-HCl buffer (20 mL) were incubated at 30, 40, 50 or 60°C. Samples (0.12 g wet free cells or 1.36 g immobilized beads) were withdrawn at specified time intervals and cooled to 30°C. The residual activity of biocatalysts was determined using the activity test described above. Experiments were conducted in duplicate.

pH Stability: 0.6 g wet free cells or 6.8 g immobilized beads (approximate 0.10 g-dw cells) were incubated in TEA-HCl buffer (20 mL) at various pH values (pH 3.0-8.0) at 30°C. Samples (0.12 g wet free cells or 1.36 g immobilized beads) were withdrawn at specified time intervals and washed with TEA-HCl buffer (100 mmol/L, pH 5.0), and the residual activity was determined as above. Experiments were carried out in duplicate.

Solvent Tolerance: 0.6 g wet free cells or 6.8 g immobilized beads (approximate 0.10 g-dw cells) were incubated in 10 mL ethyl acetate (Lg*P* 0.68), n-hexane (Lg*P* 3.5) or C_4_MIM·PF_6_ at 30°C. Samples (0.12 g wet free cells or 1.36 g immobilized beads) were withdrawn at specified time intervals and washed with TEA-HCl buffer (100 mmol/L, pH 5.0), then the residual activity was determined as above. Experiments were carried out in duplicate.

Storage Stability: 1.2 g wet free cells or 13 g immobilized beads (approximate 0.20 g-dw cells) were incubated in TEA-HCl buffer (100 mmol/L, pH 5.0) with 100 mmol/L isopropanol and stored at 4°C. Samples (0.12 g wet free cells or 1.36 g immobilized biocatalyst) were withdrawn at specified time intervals and the residual activity was determined. Experiments were carried out in duplicate.

### Scanning electron microscopy (SEM)

The immobilized biocatalyst and free cells were washed with distilled water and fixed in a 3% (w/v) glutaraldehyde solution for 2 h at room temperature. Then the fixed beads and cells were washed with distilled water to remove residual glutaraldehyde. The resulting samples and cells were successive immersed in 30, 50, 70, 90 and 100% (v/v) ethanol solutions (10 min in each solution) to remove the water from the samples. Finally, all samples were freeze-dried and the dried beads were cut to expose the interior. Beads and free cells were coated with gold via the scanning electron microscopy (Hitachi S-3700 N, Japan).

### Mass transfer experiments

To investigate the external mass transfer from the bulk solution into the beads, 50 mL Erlenmeyers flasks were used to mix 2 g Ca-alginate and alginate-chitosan beads with 6 mL 4′-chloroacetophenone (5 mmol/L) or (*R*)-1-(4-chlorophenyl)ethanol (5 mmol/L). The temperature was 30°C and the agitation was fixed at 160 rpm. The decreasing concentration of 4′-chloroacetophenone or (*R*)-1-(4-chlorophenyl)ethanol in the bulk solution were determined by GC.

And to investigate the internal mass transfer in the beads, 50 mL Erlenmeyers flasks were used to mix 2.76 g immobilized beads or 0.34 g wet cells with 8 mL buffer (100 mmol/L, pH 5.0) containing 130 mmol/L of isopropanol (the source of reducing equivalents) and 5.0 mmol/L of 4′-chloroacetophenone, capped with a septum and incubated in a shaking incubator at 30°C and 160 rpm for 120 min. The reduction of 4′-chloroacetophenone was monitored by GC. The initial reaction rate “v_0_” and kinetic constant “k” was determined using ORIGIN 7.5 (OriginLab, 2005).

### Analytical methods

Reaction mixture were assayed with a GC 2010 gas chromatograph (Shimadzu Corp., Kyoto, Japan) equipped with an HP-Chiral column (30 m × 0.25 mm, coating thickness 0.25 *μ*m, J&W Scientific, USA) and a flame ionization detector. The column temperature was held at 140°C for 9 min. Nitrogen was used as the carrier gas at a flow rate of 3 mL/min. The split ratio was 1:100 (v/v). The injector and the detector temperatures were set at 250°C and 250°C, respectively. The retention times for 4′-chloroacetophenone, 3′-methoxyacetophenone, (*R*)-1-(4-chlorophenyl)ethanol and (*S*)-1-(4-chlorophenyl)ethanol were 4.74 min, 6.20 min, 7.60 min and 8.02 min, respectively. The average error for this determination was less than 1%. All reported data are averages of experiments performed at least in duplicate.

The [α]_D_ of the reaction product, which was measured by a PerkinElmer 241 polarimeter, was compared with the [α]_D_ +49.9° (C = 2.0, ether) [43] of enantiomerically pure (*R*)-1-(4-chlorophenyl)ethanol and the product was confirmed to be (*R*)-1-(4-chlorophenyl)ethanol {[α]_D_^27^ +49.6° (C = 0.37, ether)}.

The NMR spectra of product were obtained on a Bruker DRX 300 MHz spectrometer operating at 300 MHz for ^1^ H NMR in CDCl_3_. ^1^ H NMR (300 MHz, CDCl_3_) 7.34-7.26 (m, 4 H), 4.88 (q, *J* = 6.48 Hz, 1 H), 1.97 (s, 1 H), 1.45(d, *J* = 6.81 Hz, 3 H). The result was confirmed with (*R*)-1-(4-chlorophenyl) ethanol.

## Competing interests

The authors declare that they have no competing interests.

## Authors’ contributions

WYL and MHZ participated in the design of the study; XHC carried out experiments and wrote the draft of manuscript; XTW and YL assisted to carry out experiments; TJS, HW and XDC assisted with data interpretation and revision of the manuscript. All authors read and approved the final manuscript.
